# Headspace Solid-Phase Microextraction Gas Chromatography-Mass Spectrometry and Gas Chromatography-Olfactometry Analysis of Volatile Compounds in Pineapple Breads

**DOI:** 10.3390/molecules171213795

**Published:** 2012-11-22

**Authors:** Saw Ying, Ola Lasekan, Kalla Reddi Mohan Naidu, Seye Lasekan

**Affiliations:** 1 Department of Food Technology, Faculty of Food Science and Technology, University Putra Malaysia, UPM, 43400, Serdang, Malaysia; 2 Department of Food Science, Faculty of Food Science and Technology, University Putra Malaysia, UPM, 43400, Serdang, Malaysia

**Keywords:** pineapple bread, conventionally baked, fully frozen baked, partially baked, volatiles

## Abstract

Sensorial analysis of pineapple breads (conventionally baked, Cpb; fully baked frozen, Fpb and partially baked, Ppb) showed no significant differences in terms of aroma and taste. On the contrary, the scores for the overall quality between the partially baked and conventionally baked breads showed significant (*p* < 0.05) differences. At the same time, headspace analysis using a solid-phase microextraction (SPME) method identified 59 volatile compounds. The results of the aroma extracts dilution analysis (AEDA) revealed 19 most odour-active compounds with FD factors in the range of 32–128 as the key odourants of the pineapple breads. Further analysis of the similarities and differences between the pineapple breads in terms of the key odourants were carried out by the application of PLS-DA and PLS-regression coefficients. Results showed that Ppb exhibited strong positive correlations with most of the volatile- and non-volatile compounds, while the Cpb showed significant positive correlations with hexanal and 4-hydroxy-2,5-dimethyl-3(2*H*)-furanone, and the Fpb had strong positive correlations with lactic acid, benzoic acid, benzaldehyde and ethyl propanoate.

## 1. Introduction

Bread quality is normally defined by its volume, texture, colour and flavour. However, the characteristic aroma of bread is undoubtedly one of the most important parameters influencing its acceptance by consumers [1]. Bread aroma has been widely studied and many methods to identify the compounds responsible for its flavour have been developed [2]. The flavour of bread is a result of the interaction of many factors, and depends on a large number of compounds, the majority of them with different olfactive characteristics. More than 540 compounds have been described in the complex aromatic fraction of bread [3]. All these compounds do not have same degree of influence on bread flavour [4]. Quantitatively, the most important groups are the alcohols, aldehydes, esters, ketones, acids, pyrazines and the pyrrolines [2,5].

On the one hand, the type of flour and other ingredients could influence the flavour [6], and on the other, the type of fermentation can also significantly influence bread flavour. Finally, the production process also has a vital influence on the flavour [7]. Flavour chemists are now showing more interest in volatile compounds which contribute significantly to the overall aroma of bread. Different extraction methods and analytical techniques have been employed with the aim of identifying these volatile compounds.

Several extraction techniques have been used, ranging from solvent extraction [8], distillation [3] to vacuum distillation [9]. Bread flavour components were then characterized by coupling headspace measurements with olfactometric methods such as aroma extracts dilution analysis (AEDA) [10], Solid-phase microextraction (SPME) is currently widely employed in food analysis. SPME has become one of the preferred techniques in aroma analysis, offering solvent free, rapid sampling with low cost and easy preparation. It is sensitive, selective and compatible with low detection limits [11,12,13].

In recent times, many types of breads supplemented with various nutritious, protective and filler substances have been gaining popularity worldwide [14]. The desire of consumers for healthier and fresher foods calls for a concerted effort on the part of food scientists to address such needs. Pineapple breads produced by replacing completely table sugar with concentrated pineapple juice [15] have become very popular among local communities. Pineapple juice contains high contents of trace elements (K, P, Ca, Na, Fe and vitamins C and A) [16]. Due to an increasing consumer demand for fresh bread, new technologies have been developed to offer consumers fresh bread at all times. Among these is the application of the “green dough” technology [11], in which the dough is first frozen before proofing. A first partial baking stage can also be added to the process, followed by refrigeration and storage at room temperature, or by storage under freezing conditions. These technologies produce partially baked bread and partially baked frozen bread, respectively [11]. These different breads are important in terms of innovation and they offer fresh bread after a simple final baking stage at retail outlets. The present research aims to study the composition of volatile- and non-volatile constituents in pineapple breads (*i.e.*, conventionally baked, fully baked frozen and partially baked) using HS-SMPE coupled with GC-MS/GC-O.

## 2. Results and Discussion

### 2.1. Calibration, Linearity and Recovery

The quality parameters (linearity range, LOD and recovery %) of the major volatile compounds quantified from the pineapple breads are shown in Table 1. The recoveries were good (>87.8%). This was followed by an excellent linearity in all cases. The limits of detection (LOD) which is the lowest concentration at which the results still satisfy some predetermined acceptance criteria or the mean blank value plus (3 × the relative standard deviation of the analytical blank values) [17] were low enough to determine the aroma compounds in the pineapple breads.

**Table 1:** Major volatile compounds and their performance characteristics quantified from pineapple bread.

Compounds No.	Linear range (µg.g^−1^)	Limit of detection (LOD, µg.g^−1^)	Recovery (%)
1. Acetic acid	10–200	5.92	97.4
2. Propanoic acid	5–250	1.27	98.1
3. Hexanoic acid	20–250	9.81	90.4
4. Benzoic acid	10–250	8.65	89.4
5. Ethanol	10–200	5.82	87.8
6. 2/3-Methyl-1-butanol	10–150	7.14	98.5
7. 2-Phenylethanol	10–200	4.53	94.8
8. Hexanal	10–200	7.34	90.1
9. Benzaldehyde	10–250	6.33	95.3
10. (*E,E*)-2,4-decadienal	10–150	5.98	91.4
11. 2,3-Butanedione	10–250	6.72	97.4
12. 3-Hydroxy-2-butanone	10–250	6.32	96.2
13. Ethyl acetate	10–200	7.4	99.1
14. Ethyl propanoate	10–200	9.13	92.7
15. Methyl ethanoate	10–250	4.37	96.3
16. Ethyl octanoate	10–200	7.09	90.4
17. Furfural	10–200	5.97	90.6
18. Furfury alcohol	10–250	6.54	94.4
19. 2-Methylpropanoic acid	10–200	8.35	90.8
20. 2,3-Dihydroxy-6-methyl-4*H*-pyran-4 one	10–250	7.66	91.3
21. 4-Hydroxy-2,5-dimethyl-3(2*H*)-furanone	10–200	5.78	96.7

### 2.2. Sensory Evaluation of Breads

The sensory evaluation showed appreciable differences among the different pineapple bread types (Table 2). The best results were obtained in the case of the partially baked pineapple bread (Ppb), while the conventionally baked pineapple bread (Cpb) recorded the lowest scores. Statistical analysis (one-way ANOVA) showed that the scores for aroma and taste between all types of pineapple breads were not significantly different (*p* > 0.05). On the contrary, the scores for the overall quality between partially baked pineapple bread and conventionally baked bread showed significant (*p* < 0.05) differences. The overall quality is that sensational attributes that makes a product fundamentally different from others. Some of the factors contributing towards quality of food are; appearance, colour, taste, aroma, adulterants and nutritional value. While the Ppb produced higher intensity of dough and sourdough flavour with slight fruity notes, the Cpb and Fpb had lower flavour nuances.

**Table 2:** Panellists’ sensory evaluation of pineapple bread samples (CPB, FPB and PPB).

Attributes	Bread type *		
	CPb	FPb	PPb
Aroma	7.1 ± 0.1 ^ab^	7.6 ± 0.1 ^a^	8.0 ± 0.2 ^a^
Taste	7.4 ± 0.2 ^a^	8.0 ± 0.1 ^a^	8.4 ± 0.1 ^a^
Overall quality	7.0 ± 0.1 ^b^	8.3 ± 0.1 ^a^	8.7 ± 0.1 ^a^

* Mean ± SD with the same superscripts are not significantly (*p* > 0.05) different; CPb: Conventional pineapple bread, FPb: Fully frozen pineapple bread, PPb: Partially baked pineapple bread.

### 2.3. Identification of Volatile Compounds

To elucidate the molecular principles responsible for the observed aroma impressions, the flavour volatile compounds of the pineapple breads were first isolated by means of headspace solid-phase microextraction and were subsequently analysed by means of gas chromatography-olfactometry, as well as mass spectrometry. This method and the application of the aroma extract dilution analysis (AEDA) led to the detection of 59 odour-active compounds in the FD factor range of 2–128. These compounds, together with their odour impressions and retention indices are shown in Table 3.

The complex chemical and enzymatic reactions which occurred during fermentation and baking gave rise to a number of volatile and non-volatile compounds. It is likely that these compounds in no doubt contributed to the overall flavour of the final pineapple bread. In this study, the identified volatile compounds comprised eight acids, eight alcohols, 12 aldehydes, 12 ketones, 10 esters and three furan derivatives (Table 3). In general, all main classes of compounds commonly listed as thermally generated flavours in baked breads were identified in the pineapple breads (Cpb, Fpb and Ppb). The flavour compounds revealed fruity (nos. 1, 5, 8, 15, 17, 26, 46, 56, and 58), buttery (nos. 4, 7 and 45), roasty (nos. 29, 35 and 37), sweaty (nos. 14, 23, 28 and 50), fatty (nos. 27, 33, 42, 43, 44, 48 and 54) odour qualities. Also, diverse sweet, malty, vanilla- or honey-like/flowery compounds were detected (nos. 9, 18, 25, 30, 36, 38, 49, 57 and 59) (Table 3). It is noteworthy that aroma compound number 20 (Table 3) with popcorn-like aroma failed to yield any signal at the flame ionization detector. This indicated a very low odour threshold for this compound. This compound was later characterized by co-injection with the reference compound on the DB-5 column (Table 3) and its odour quality at the sniffing port revealed it to be 2-acetyl-1-pyrroline.

**Table 3:** Volatile compounds identified in pineapple breads (conventionally processed, CPb; fully frozen, Fpb and partially baked, Ppb).

Number	Odourant ^a^	Odour quality ^b^	FD	Retention index DB-5	Previously identified in bread ^c^
1	Ethyl acetate	Pineapple-like	32	628	2, 4
2	Propanoic acid	Rancid	32	668	4, 7
3	Methyl-2-methylpropionate	Fruity, sweet	4	687	9
4	2,3-Pentanedione	Buttery	16	710	1, 4, 5 & 10
5	Ethyl propionate	Fruity	64	713	
6	Butanoic acid	sour	2	718	4, 5
7	3-Hydroxy-2-butanone	Buttery	64	720	2, 3
8	Ethyl-2-methyl propionate	Sweet-fruity	4	754	4, 6, 9
9	2-/3Methyl-1-butanol	Malty	128	769	2, 3, 4
10	1-Hexen-3-one	Green	2	774	1, 4, 10
11	Hexanal	Green, tallow	32	787	1,2, 3, 7
12	2,3-Butanediol	Fruity, onion	8	802	1, 2
13	Furfural	Bread-like	64	826	1, 2, 3
14	2-Methyl butanoic acid	Sweaty	16	831	1, 4
15	Ethyl-2-methyl butanoate	Fruity	8	838	9
16	Furfuryl alcohol	Burnt	16	863	
17	Methyl ethanoate	Fruity	32	864	4
18	(*Z*)-4-Heptenal	Biscuit-like, sweet	16	894	1, 4, 5, 6, 7, 10
19	Methional	cooked-potato	4	919	4, 5, 6, 7, 10
20	2-Acetyl-1-pyrroline ^d^	Popcorn-like	16	922	4, 5, 6, 10
21	Benzaldehyde	Almond-like	64	936	4,7
22	Ethanol	Alcoholic	32	945	3, 6,
23	Hexanoic acid	Sweaty	32	961	1, 3, 4
24	1-Octen-3-one	Mushroom-like	4	970	1, 4, 5, 10
25	(*Z*)-1,5-Octadien-3-one	Geranium-like	2	987	4, 5, 7, 9
26	Ethyl hexanoate	Fruity (apple)	8	1002	1, 2, 9
27	Octanal	Soapy, fatty	4	1006	1, 4, 9
28	4-Hydroxybutanoic acid	Sweaty	16	1018	
29	Acetylpyrazine	Toasty	4	1030	1, 4, 7, 10
30	Phenylacetaldehyde	Honey-like	16	1042	2, 4, 5, 7
31	Tetrahydro-6-methyl-2 *H*-pyran-2-one	Coconut	2	1050	1,2
32	4-Methoxy-2,5-dimethyl-3 (2 *H*)-furanone	Caramel-like	16	1057	9
33	(*E*)-2-Octenal	Nutty, fatty	8	1062	1, 4, 5, 6, 7
34	4-Hydroxy-2,5-dimethyl-3(2 *H*)-furanone	Sweet, caramel	32	1022	2, 4, 9
35	2-Ethyl-3,5-dimethylpyrazine	Roasty	4	1080	4, 5, 7, 10
36	Linalool	Flowery	4	1092	1, 2
37	2-acetyl-2-thiazoline	Roasty	8	1108	4
38	2-Phenylethanol	Honey	128	1136	1, 2, 3, 4, 10
39	2-Propionyl-2-thiazole	Roasty	2	1125	4, 10
40	2,3-Dihydroxy-6-methyl-4 *H*-pyran-4-one	Caramel-sweet	64	1141	8
41	2,3-Diethyl-5-methylpyrazine	Earthy	4	1157	4, 7, 10
42	(*E*)-2-Nonenal	Fatty, green	4	1164	4, 5, 6, 7
43	Ethyl octanoate	Fruity, fatty	32	1201	1, 5
44	(*E,E*)-2,4-Nonadienal	Fatty	2	1220	1, 4, 5, 6
45	2-Methylpropanoic acid	Buttery, rancid	32	1221	4
46	Ethyl phenyl acetate	Fruity, sweet	8	1253	1
47	*Y*-Octalactone	Coconut-like	16	1261	1, 9
48	Decanol	Fatty	4	1269	1
49	2-Phenylacetic acid	Honey	8	1270	4
50	Benzoic acid	Sweaty	32	1287	
51	δ-Octalactone	Coconut-like	2	1288	1, 2, 9
52	Butyrolactone	Caramel, sweet	4	1299	2
53	4-Vinylguaiacol	Curry, clove	4	1310	1, 10
54	(*E,E*)-2,4-Decadienal	Fatty	32	1313	1, 4, 5, 6
55	α-Copaene	Wood	2	1393	1
56	β-Damascenone	Sweet, fruity	8	1393	1, 4, 7, 9
57	Vanillin	Vanilla-like	16	1410	1, 4, 9
58	*Y*-Decalactone	Fruity, peach-like	4	1473	9
59	Ethyl octadecanoate	Flowery	4	2205	1

^a ^The compounds were identified by comparing them with reference substances on the basis of the following criteria: retention index on DB-5 as given in the table, mass spectra obtained by MS (EI), and odour quality as well as odour intensity perceived at the sniffing port. ^b^ Odour quality perceived at the sniffing port. ^c^ Reported in the literature as volatile compounds of bread in: ^1 ^Adams [18]; ^2^ Elss *et al*. [19]; ^3^ Jensen *et al*. [20]; ^4^ Pozo-Bayon *et al*. [1]; ^5 ^Rychlik & Grosch [8]; ^6^ Schieberle & Grosch [5]; ^7^ Schieberle & Grosch [21]; ^8^ Silva *et al*. [22]; ^9^ Tokitomo *et al*. [23]; ^10^ Zehentbauer & Grosch [24]. ^d^ The MS signal was too weak. The compound was later characterized by co-injection with reference compounds.

The production of volatile compounds is generally influenced by dough fermentation [24], proofing and baking [25]. For instance, prolonged dough fermentation has been reported to increase the concentration of some volatile compounds such as 3-methylbutanol, 2-phenylethanol and ethanol [26]. These compounds are directly linked to the fermentative activity of the yeast in the dough fermentation step [27]. In the present study, compounds with appreciably high concentrations were ethanol, ethyl acetate, 2,3-butanedione, 3-hydroxy-2-butanone and ethyl propionate (Table 4). Interestingly, the partially baked pineapple bread (Ppb) recorded the highest concentration of odourants in all cases (Table 4). This is probably due to the slightly higher fermentation time of the Ppb, which might have produced appreciable amounts of free amino acids. The free amino acids can act as precursors of the Strecker reaction leading to the increases obtained in odourant’s concentration [26].

**Table 4:** Most odour-active volatile compounds (FD > 32) identified in pineapple bread samples (Cpb, Fpb and Ppb) *.

Compound	CPb	Fpb	Ppb
**ACIDS**			
Propanoic acid	0.03 ± 0.0	0.02 ± 0.0	0.35 ± 0.0
2-Methylpropanoic acid	0.07 ± 0.0	0.07 ± 0.0	0.14 ± 0.0
Hexanoic acid	0.03 ± 0.0	0.03 ± 0.0	0.11 ± 0.0
Benzoic acid	0.30 ± 0.01	0.33 ± 0.01	0.45 ± 0.01
**ALCOHOLS**			
Ethanol	2.16 ± 0.01	2.14 ± 0.02	2.35 ± 0.01
2/3-Methyl-1-butanol	0.15 ± 0.0	0.16 ± 0.0	1.67 ± 0.01
2-Phenylethanol	0.18 ± 0.0	0.18 ± 0.0	0.89 ± 0.01
**ALDEHYDES**			
Hexanal	0.53 ± 0.0	0.51 ± 0.01	0.50 ± 0.02
Benzaldehyde	0.71 ± 0.01	0.73 ± 0.02	0.68 ± 0.01
(*E,E*)-2,4-Decadienal	1.03 ± 0.2	1.03 ± 0.07	1.14 ± 0.02
**KETONES**			
2,3-Butanedione	1.76 ± 0.06	1.75 ± 0.03	2.04 ± 0.12
3-Hydroxy-2-butanone	1.77 ± 0.03	1.79 ± 0.01	1.87 ± 0.11
2,3-Dihydroxy-6-methyl-4 *H*-Pyran-4-one	0.04 ± 0.0	0.05 ± 0.0	1.35 ± 0.1
**ESTERS**			
Ethyl acetate	4.28 ± 0.3	4.29 ± 0.5	5.28 ± 0.2
Ethyl propionate	1.63 ± 0.02	1.60 ± 0.02	1.57 ± 0.01
Methyl ethanoate	0.06 ± 0.0	0.06 ± 0.0	0.05 ± 0.0
Ethyl octanoate	1.05 ± 0.06	1.05 ± 0.0	1.06 ± 0.0
**FURANS**			
4-Hydroxy-2,5-dimethyl-3(2 *H*)-furanone	1.33 ± 0.6	1.37 ± 0.1	1.35 ± 0.1
Furfural	0.93 ± 0.01	1.01 ± 0.0	1.95 ± 0.1
**NON-VOLATILES**			
Lactic acid (µg/g)	37.1 ± 2.5	38.7 ± 1.0	54.2 ± 5.7
TTA ^1^	1.42 ± 0.1	1.44 ± 0.2	2.03 ± 0.1

* µg equivalents of 1-butanol per gram of bread. ^1^ mL NaOH 0.1 N/10 g of bread.

### 2.4. Multivariate Analysis

**Figure 1:**
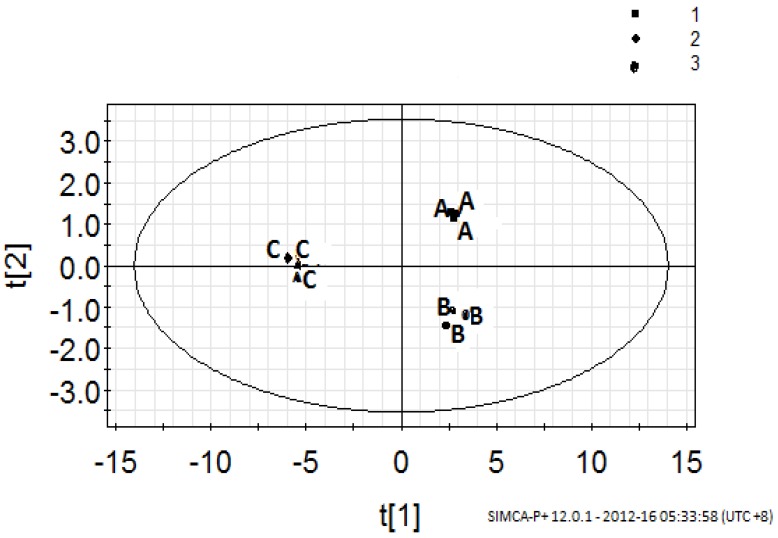
Score scatter plot of PLS-DA to show the similarities and differences between the pineapple breads. A: Conventionally baked (Cpb); B: Fully frozen baked (Fpb) and C: Partially baked (Ppb).

In order to examine the similarities and differences between the three pineapple breads (Cpb, Fpb and Ppb) in terms of the volatile compounds associated with each sample, principal component analysis (PCA) was employed. PCA provided easy visualisation of the complete data set in a reduced dimension plot, showing variability between volatile- and non-volatile compounds of breads. This method was employed to establish relationships between pineapple breads (Cpb, Fpb and Ppb) and their flavour volatile compounds (Table 4). Based on the bread samples’ grouping from PCA, a partial least square discriminant analysis (PLS-DA) was established (Figure 1). The scatter plot of scores of the first two components (in the PLS-DA which explained 92.53% of the total variance in the data) showed the distinctions among the different pineapple breads.

**Figure 2:**
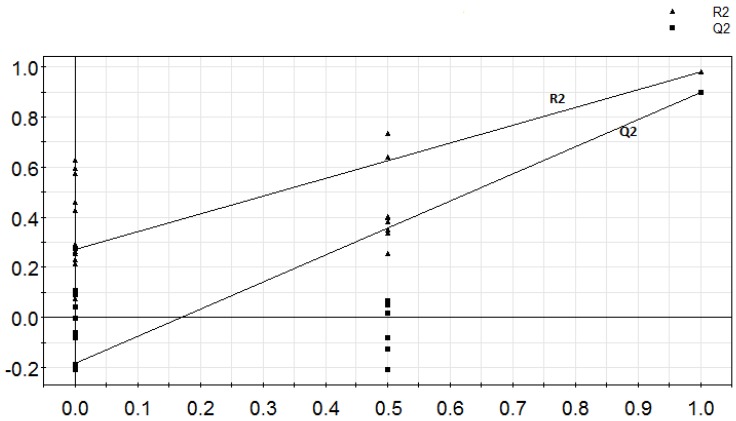
The validation plot for PLS and PLS-DA models. Intercepts: R2 = (0.0, 0.271), Q2 = (0.0, −0.182).

The PLS-DA model (which was based on three replicates and three breads) was however, validated using the response of permutation test through 100 permutations (Figure 2). A model is said to be valid when the intercept of R2 is <0.3 and intercept of Q2 is <0.05 [25]. In this study, the R2 is 0.271 and Q2 is −0.182 respectively. This plot (Figure 2) strongly indicates that the original model is valid because the regression line of the Q2-point intersects the vertical axis on the left and below zero.

**Figure 3:**
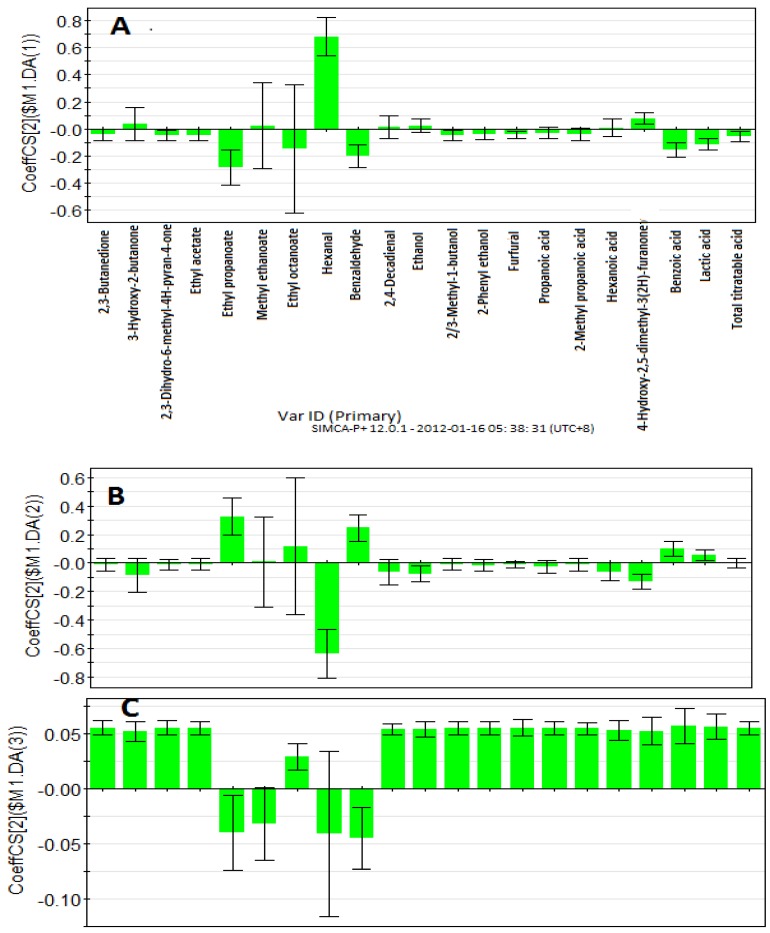
The regression coefficients of scaled and centered variables for the response components. A: Cpb; B: Fpb and C: Ppb.

Pineapple breads (Cpb, Fpb and Ppb) were separated according to their processing methods (Figure 1). While conventionally baked pineapple bread (Cpb) was situated in the area of positive components 1 and 2, the fully frozen baked pineapple bread (Fpb) was within the area of positive component 1 and negative component 2 respectively. However, partially baked pineapple bread (Ppb) was situated in the area of negative component 1 and positive component 2 respectively. The inter-relationship between the pineapple breads (Cpb, Fpb and Ppb) and the volatile- and non-volatile compounds were examined by the PLS-regression coefficients of scaled and centred variables (Figure 3A–C). Meanwhile, the Cpb showed significant (*p* < 0.05) positive correlation with hexanal and 4-hydroxy-2,5-dimethyl-3(2*H*)-furanone, it however, revealed strong negative correlations with benzoic acid, benzaldehyde, and ethyl propionate (Figure 3A). On the other hand, Fpb, revealed strong positive correlations with lactic acid, benzoic acid, benzaldehyde and ethyl propionate (Figure 3B). Moreover, the Ppb showed positive correlations with most of the volatile- and non-volatile compounds respectively (Figure 3C). It however, showed negative correlations with only two compounds namely; benzaldehyde and ethyl propionate. As can be seen, while the alcohols (ethanol, 2/3-methyl-1-butanol and 2-phenylethanol) and the acids showed high positive correlations in the partially baked pineapple bread (Ppb), they however, revealed negative correlations in both conventional and fully frozen baked pineapple breads (Figure 3A–C). Previous studies on sourdoughs [26,27,28] have shown that the presences of acids in sourdoughs are positively valued, as they strengthen the flavour.

As the conventional pineapple bread (Cpb) and the fully frozen pineapple bread (Fpb) were based on the same recipe and fermentation regime, the differences identified in their volatile compounds may be linked to the freezing and thawing stages that distinguished them.

## 3. Experimental

### 3.1. Materials

All experiments were carried out with flour purchased from a local market in Malaysia. Its composition was: 12% protein, 13.5% moisture basis and 77% starch (determined by a Megazyme starch kit, Megazyme Int., Wicklow, Ireland), of which 5% was damaged starch (determined by a Megazyme damaged starch kit). Fresh pineapple fruits (*Ananas comosus* L) of Josephine cultivar with similar characteristics of ripening (skin colour, flat eyes and Brix) were used.

### 3.2. Chemicals

The following odourants: 97% 2-methyl-1-butanol, 96% 3-methyl-1-butanol, 98% 2-phenyl ethanol, 98% 1-butanol, 96% acetic acid, 98% propanoic acid, 97% hexanoic acid, 96% 4-hydroxy-butanoic acid, 89% benzoic acid, 97% hexanal, 98% benzaldehyde, 98% (*E,E*)-2,4-decadienal, 97% furfural, 98% furfuryl alcohol, 98% ethyl acetate, 98% ethyl propanoate, 97% methyl ethanoate, 97% ethyl octanoate, 98% 2-methylpropanoic acid, 96%; were obtained from (Aldrich, Steinheim, Germany); 99% 2,3-butanedione, 98% 4-hydroxy-2,5-dimethyl-3(2*H*)-furanone, 98% 3-hydroxy-2-butanone were purchased from Fluka (Neu-Ulm, Germany). Stock standard solutions of 10^3^ or 10^4^ mg L^−1^ of each component were prepared by dissolving the pure standard in 40% (v/v) ethanol. The samples were stored at 4 °C. Working standard solutions were prepared daily by mixing an aliquot of each individual solution and diluting with ultra pure water (Millipore Co., Bedford, MA, USA) to obtain a desired concentration. 

### 3.3. Sample Preparation

#### Preparation of Pineapple Juice Concentrates

Fresh pineapple fruits were washed in cold tap water and drained. They were manually cut up and the juice extracted in a Panasonic juice extractor (MJ-7 juice extractor, Panasonic, Bracknell, U.K). The juice (1,000 mL) obtained was filtered, dispensed in a beaker and replaced on the centre of a programmable domestic Panasonic microwave oven (Model NN 573-MF) with maximum output of 700 W at 2,450 MHz The oven has adjustable power (wattage) and time controllers, and was fitted with a turntable. Heating was carried out at 350 W for 23 min.

### 3.4. Bread Preparation

**Figure 4:**
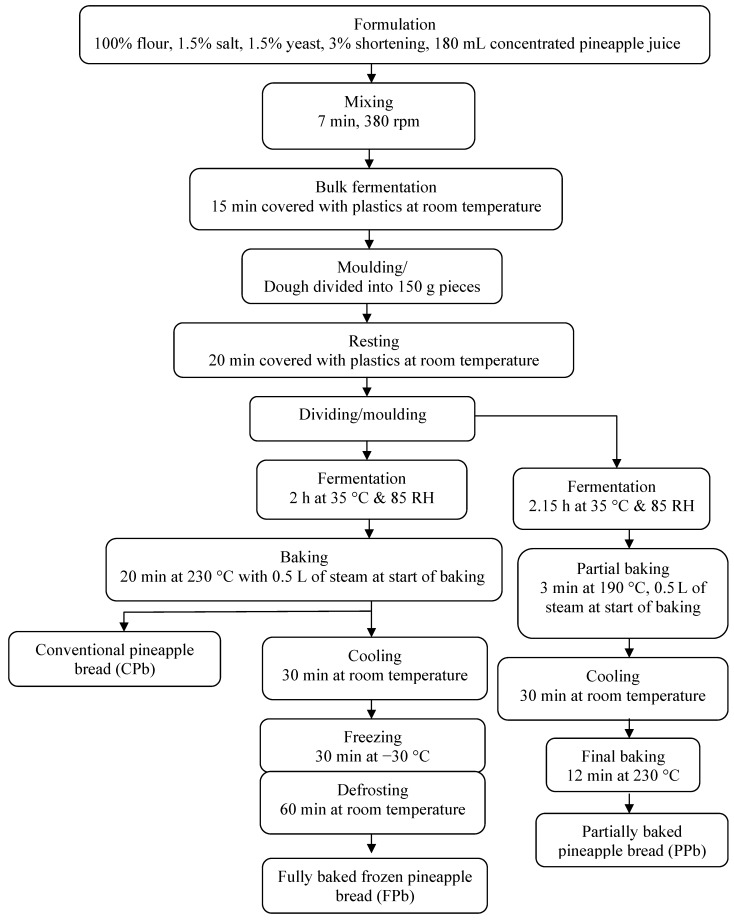
Flow sheet on the fermentation steps and the production procedure for the different pineapple breads (CPb, FPb and PPb).

The recipes and the production processes of the pineapple breads are as shown in Figure 4 [15]. For the preparation of the pineapple breads, three different methods (conventional, fully baked and frozen and partially baked) were employed. The dough was mixed in a KN-200 mixer (Taisho, Denki Co., Ltd., Shiga, Japan) for 7 min at 380 rpm. The dough was flattened with a roller to obtain a round, symmetrical and homogenous thick sheet. It was then divided into 20 samples of 150 g pieces and allowed to rest for 20 min at room temperature. The dough pieces were divided into two batches (*i.e.*, 10 samples of 150 g pieces).

One batch was fermented for 2 h at 35 °C and 85 relative humidity (RH) while the second batch was fermented for 2.15 h at 35 °C and 85 RH. After fermentation, the first batch was baked at 230 °C for 20 min with an initial 0.5 L steam at the start of baking in a ventilated oven. Baked samples were further divided into two groups. A group is referred to as the conventional pineapple bread (Cpb) while the other group was frozen at −30 °C for 30 min and latter defrosted at room temperature for 60 min. This group is referred to as the fully baked frozen pineapple bread (Fpb).

The second fermented batch was partially baked at 190 °C for 3 min with 0.5 L steam at the start of baking similarly to the first batch. The baked bread sample was cooled for 30 min at room temperature and final baking was carried out in a static oven at 230 °C for 12 min. This sample is referred to as partially baked pineapple bread (Ppb).

### 3.5. Sensory

The sensory acceptability of the pineapple breads (Cpb, Fpb and Ppb) were determined using 24 University Putra Malaysia students whose ages ranged from 19 to 30 years. The students were made up of 12 females and 12 males, respectively. The samples were coded with a three-digit randomised number. The panellists were provided with water as mouth rinse. Each partitioned booth in the taste panel section was lighted with natural white fluorescent light. Panellist scored the breads according to a preference protocol including a scale from 0 (unacceptable) to 10 (excellent) [29]. Odour intensity was monitored using an odour intensity scale, which provides a verbal description of an odour sensation to which a numerical value is assigned (*i.e.*, 0 = no odour; 1 = very weak; 2 = weak; 3 = distinct; 4 = strong; 5 = very strong and 6 = intolerable) [7].

### 3.6. Acidity Analysis

Total titratable acidity (TTA) was measured using the method described by Martinez-Anaya and others [30] and lactic acid was determined using the method described by Quilez and other [31].

### 3.7. Headspace Solid-Phase Microextraction (HS-SPME)

A cross sectional cut (10 g of each bread samples) obtained from the central part was mixed together with NaCl (10 mL, 1 g·mL^−1^) and shaken for 30 s. Five grams of this mixture was placed in a 125 mL flask with a magnetic stirring bar and 5 µL of internal standard and closed with cap. The flask was incubated for 60 min in a heated tray oven at 40 °C. An SPME fibre with two different coatings (75 µm carboxen^TM ^/polydimethylsiloxane; CAR/PDMS stablFlex^TM^) (Supelco Co., Bellefonte, PA, USA) was inserted in the headspace of the vial for 10 min to collect the volatiles. The type of fibre used can often affect the selectivity of the extraction and by using a mixed type; good selectivity is obtained for non-polar analyte as well as for polar analyte. This type of fibre was used successfully in the past for the characterisation of the aroma compounds of partially baked bread [11]. After each extraction, the fibre was inserted into the GC injector port using a 0.75 mm i.d liner (in order to improve the GC resolution). Desorption time and temperature were 5 min and 250 °C respectively. All experiments and sample measurements were carried out in triplicate and the average values were recorded.

### 3.8. GC-MS Analysis

The identification and quantification of volatile compounds was carried out with a Shimadzu (Kyoto, Japan) QP-5050A GC-MS instrument equipped with a GC-17A Ver. 3 gas chromatograph with a flame ionization detector (FID). The column was a DB-5 column (30 m × 0.32 mm i.d., film thickness 0.25 µm J & W Scientific, Folsom, CA, USA). Helium was used as carrier gas at a flow rate of 1.5 mL min^−1^, injection temperature, 250 °C; detector temperature, 280 °C; temperature program commenced at 50 °C and held for 3 min, then raised to 250 °C at a rate of 15 °C min^−1^, held for 10 min and then increased to 280 °C at a rate of 10 °C min^−1^, with a final hold time of 5 min. The effluent from the capillary column was split into 2:1 (by vol.) onto two uncoated but deactivated fused silica capillaries (50 cm × 0.32 mm) leading to a FID and a sniffing port.

The mass spectrometer was operated in electron impact mode with the following conditions. The source temperature was 250 °C; the quadruple temperature selected was 280 °C and the relative electron multiplier voltage (EM) applied was 400 V with a resulting voltage of 1,553 V. In order to improve the detection limits, the selected ion monitoring (SIM) mode was used. Compounds identification was based on comparison of linear retention indices (RI), mass spectra (comparison with standard MS spectra databases: Wiley 6), and injection of standards. The quantification was performed using 1-butanol as the added standard. The concentrations of volatile compounds were expressed in microgram equivalents of 1-butanol per gram of bread.

### 3.9. GC-Olfactrometry Analysis

Each bread sample (dry weight) (400 g) was mixed with water (1,800 mL) in a 4-litre distilling flask. The distilling flask was heated in a water bath at 40 °C and the volatile compounds were steam distilled under vacuum (30 mbar) for 5 h. The distillate was extracted with dichloromethane, dried with sodium sulphate and concentrated to 50 µL [26].

GC-olfactometry analysis was performed on a Thermo Scientific GC instrument (Thermo Fisher Scientific, Rivoltana, Italy) equipped with a split-splitless injector, a FID and an ODP 3 Olfactory Detector Port (Gerstel, Mulheim, Germany). At the column outlet, the eluate was split 1:1 to simultaneously detect volatile compounds by FID and sniffing. Samples (4 µL) were separated on a 60 m × 0.32 mm i.d., 0.25 µm film thickness DB-5 column (J & W Scientific) according to the same temperature programmed as reported for the GC-MS analysis. Helium was used as a carrier gas at the flow rate of 1.5 mL min^−1^. Injector and FID temperature was 250 °C; hydrogen, air and nitrogen (make up) flow rates were 30, 450 and 30 mL min^−1^ respectively. The sniffing was performed by three trained panellists, who presented satisfactory sensitivity and reproducibility and agreement with one another. For each GC-O analysis, sniffing was divided into three sessions of 20 min and panellists were asked to characterise the detectable aroma with a freely chosen descriptor.

### 3.10. Aroma Extracts Dilution Analysis (AEDA)

The FD factors of the odour-active compounds were determined by AEDA [32]. Each bread sample (200 g) was immediately frozen with liquid nitrogen and later homogenized with anhydrous sodium sulphate (200 g) in a commercial blender (Moulinette, Numberg, Germany). The powder was extracted with dichloromethane (1 L) for 8 h at 40 °C using a Soxhlet extractor. The extract was concentrated to ~100 mL by distilling off the solvent. The distillate was extracted with an aqueous sodium carbonate (0.5 mol/L, 3 × 50 mL) to remove acidic volatiles. The aqueous solution was washed with dichloromethane (50 mL), and the organic phase were combined, dried over anhydrous sodium sulphate, filtered, and concentrated to ~0.1 mL. The following series: the neutral-basic and acidic fractions were step wisely diluted with dichloromethane (1 + 1, v/v), and each dilution was analyzed by gas chromatography/olfactometry to give the flavour dilution (FD) factors of odour-active compounds.

### 3.11. Statistical Analysis

Raw data obtained from each sensory session were averaged across panellists, and the results were studied by one-way analysis of variance (ANOVA). Duncan’s multiple range tests were used to determine significance among results. Partial least square discriminant analysis (PLS-DA) and PLS-regression coefficient were chosen as an exploratory technique to describe and summarise data by grouping variables that are correlated. The multivariate statistical analyses were performed with the SIMCA-P software (v. 12.0, Umetric, Umeå, Sweden) [33] PLS regression is specifically designed to determine relationships which exist between blocks of dependent (Y, bread types) and independent (X, volatiles) variables by seeking underlying factors common to both sets of variables [24].

## 4. Conclusions

The sensory profiles for the different pineapple breads (Cpb, Fpb and Ppb) were found to be affected by processing parameters such as fermentation time, freezing and baking protocols. While the partially baked pineapple bread (Ppb) produced higher intensity of dough and sourdough like flavour with slight fruity notes, the other two pineapple breads (Cpb and Fpb) produced much lower flavour nuances. The AEDA results revealed the 19 most odour-active compounds with flavour dilution (FD) factors in the range of 32–128 as the key odourants of the pineapple breads. Further analysis of the similarities and differences between the pineapple breads in terms of the key odourants were carried out by the application of PLS-DA and PLS-regression coefficients. Results showed that Ppb exhibited strong positive correlations with most of the volatile- and non-volatile compounds, while the Cpb showed significant positive correlations with hexanal and 4-hydroxy-2,5-dimethyl-3(2*H*)-furanone, and the Fpb had strong positive correlations with lactic acid, benzoic acid, benzaldehyde and ethyl propanoate. These different pineapple breads are important products in terms of innovation and they represent processes that reduce cereal waste.
